# Optically levitated nanoparticle as a model system for stochastic bistable dynamics

**DOI:** 10.1038/ncomms15141

**Published:** 2017-05-09

**Authors:** F. Ricci, R. A. Rica, M. Spasenović, J. Gieseler, L. Rondin, L. Novotny, R. Quidant

**Affiliations:** 1 ICFO-Institut de Ciencies Fotoniques, The Barcelona Institute of Science and Technology, Castelldefels, Barcelona 08860, Spain; 2 Physics Department, Harvard University, Cambridge, Massachusetts 02138, USA; 3 ETH Zürich, Photonics Laboratory, Zürich 8093, Switzerland; 4 ICREA-Institució Catalana de Recerca i Estudis Avancats, Barcelona 08010, Spain

## Abstract

Nano-mechanical resonators have gained an increasing importance in nanotechnology owing to their contributions to both fundamental and applied science. Yet, their small dimensions and mass raises some challenges as their dynamics gets dominated by nonlinearities that degrade their performance, for instance in sensing applications. Here, we report on the precise control of the nonlinear and stochastic bistable dynamics of a levitated nanoparticle in high vacuum. We demonstrate how it can lead to efficient signal amplification schemes, including stochastic resonance. This work contributes to showing the use of levitated nanoparticles as a model system for stochastic bistable dynamics, with applications to a wide variety of fields.

## Abstract (web summary)

Nano-mechanical resonators improve with high-Q factor and light mass, but this leads to the onset of nonlinear behaviour. Here the authors demonstrate precise control of the non-linear and bistable dynamics of a levitated nanoparticle in vacuum, using it as model system to study stochastic bistable phenomena.

Nano-mechanical resonators need to meet criteria of light mass and high-Q factor in order to maximize their performances, when operated as linear force transducers. However, these features lead to intrinsically nonlinear behaviour^1^, with consequent vanishing dynamical range. To overcome this limitation, modern nanotechnology requires new sensing schemes that take nonlinearities into account and even benefit from them^2^. Many of the proposed solutions operate inside an instability region^3^,^4^ or close to a bifurcation point^5^,^6^, where the system ideally becomes infinitely sensitive. Others exploit fluctuations of noisy environments to trigger stochastic resonances^7^ that amplify weak harmonic signals^8^,^9^,^10^. For all these sensing applications, a single nanopartticle optimally decoupled from the environment represents a particularly interesting system in the family of high-Q resonators. In fact, since its very first realization^11^, optical levitation of a nano-scale object in vacuum has enabled several ground-breaking experiments, including demonstration of zeptonewton force sensitivity^1^,^12^, tests of fluctuation theorems and stochastic thermodynamics^13^,^14^, as well as the observation of photon recoil heating^15^.

In this work, we demonstrate full control on the linear, non-linear and bistable dynamics of a levitated nanoparticle in high vacuum and under the effect of external noise. The potential of our platform is validated by the implementation of two nonlinear amplification schemes, including stochastic resonance^7^. Remarkably, we demonstrate, in excellent agreement with theory, up to ∼50 dB amplification of non-resonant harmonic force at the atto-newton scale. In addition, the unprecedented level of control achieved will enable the use of levitated nanoparticles as a model system for stochastic bistable dynamics with applications to a wide range of fields including biophysics^16^,^17^, chemistry^18^,^19^ and nanotechnology^20^.

## Results

### System

**Figure 1:**
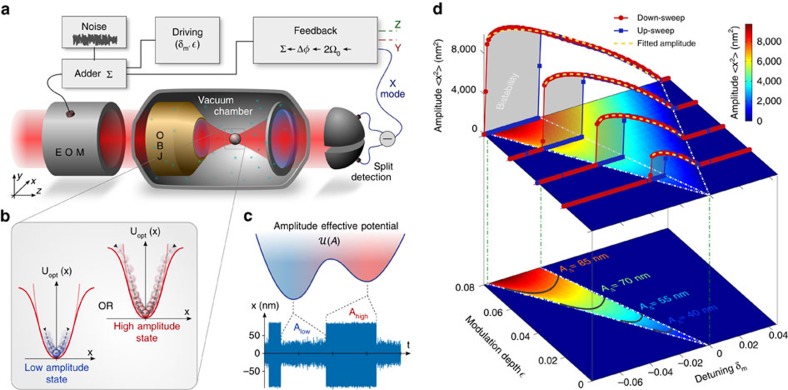
Experimental set-up and nonlinear response. (**a**) A microscope objective (OBJ) focuses a laser beam inside a vacuum chamber, where a single silica nanoparticle is trapped. Its motion is measured with a split detection scheme and parametric feedback is applied via an EOM in order to cool its center of mass motion. (**b**) A linear low amplitude and a nonlinear high amplitude oscillation states coexist when bistability is induced by the coherent driving. (**c**) The bistable dynamics can be modelled by an effective amplitude double-well potential. Injected optical noise activates stochastic switching between the two states. (**d**) (top) Measured 2D false colour map representing the particle amplitude response in the driving parameter phase space 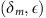. The red and blue cross-cuts, correspond respectively to down- and up- sweeps, and emphasize the typical hysteretic non-linear response of the resonator. Yellow lines are fits to the high amplitude solution *A*(*δ*_m_) and allow to retrieve the nonlinear coefficients *η* and *ξ*. The bistable regime (bottom map), can be isolated subtracting up-sweep from down-sweep maps. Solid lines correspond to iso-amplitude lines (2): subsets of the parameter space where 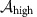, and consequently 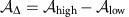, are kept constant.

Our experiment is sketched in Fig. 1a. A single silica nanoparticle (*d*∼177 nm in diameter) is optically trapped in vacuum by a tightly focused laser beam, and its 3D trajectory monitored with a balanced split detection scheme. The detector signal controls an electro-optic modulator (EOM) that feedback-cools all three translational degrees of freedom by modulating the laser intensity. In high-vacuum conditions (*P*∼10^−6^ mbar) the mechanical Q-factor, given by the gas damping *γ*_0_, is measured to be *Q*=1.2 × 10^8^. Under feedback, the nanoparticle oscillates at non-degenerate angular frequencies Ω_0_^(*x*,*y*,*z*)^ with very small amplitudes, corresponding to sub-Kelvin effective temperatures^11^. By optimizing the feedback settings (Supplementary Note 2) and carefully screening important sources of noise such as mechanical vibrations and air turbulences, we obtain a highly stable resonator with frequency fluctuations improved by one to two orders of magnitude compared to previous works^1^,^21^ (Supplementary Note 3). When one of the spatial modes is parametrically driven at resonance^21^, the particle explores the anharmonic part of the optical potential, which can be modelled as a Duffing nonlinearity^1^. As a result, the equation of motion for the driven coordinate, which we choose to be *x*, reads


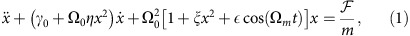


where *η* is the non-linear feedback-induced damping coefficient, *ξ* is the Duffing term prefactor, 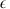 and Ω_*m*_ are, respectively, the modulation depth and modulation frequency of the driving signal. Finally, the fluctuating force 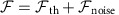 has two contributions. The first one, 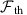, represents the stochastic force arising from random collisions with residual air molecules in the chamber, while the second, 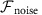, represents artificial parametric noise that we add through the EOM. See Supplementary Note 1 for further experimental set-up details.

### Amplitude response

The nonlinear response of the resonator is fully characterized by measuring the oscillation amplitude in the driving parameters phase space 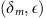, where *δ*_m_=Ω_m_/Ω_0_−2 is the normalized detuning^21^. When the modulation frequency is decreased (down-sweep) across the resonant condition *δ*_*m*_=0, the amplitude response spans a triangular region corresponding to the first instability tongue^22^. This is shown in the top 2D false colour map of Fig. 1d. In the opposite case of a frequency up-sweep, however, the system displays hysteresis, and the tongue results in a narrower width. The red and blue cross cuts in Fig. 1d clearly show this behaviour, identifying a region where two stable oscillation states coexist (Fig. 1b,c). The bistability region, shown at the bottom of Fig. 1d, is obtained by subtracting up- from down-sweeps maps. By fitting our experimental data with the analytical solution 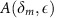 (See Supplementary equation 1; ref. 21) we retrieve the nonlinear coefficients *η*=(18.1±0.4) μm^−2^ and *ξ*=(−9.68±0.15) μm^−2^. Such precision measurements are made possible by the highly stable oscillation frequencies that lead to minimal drifts of the detuning parameter (Δ*δ*_m_≈2ΔΩ/Ω_0_⪅10^−3^, over the entire measurement time), therefore allowing to predict with excellent agreement the boundaries of the instability tongues (white lines in Fig. 1d). Further details on spectral features of the nonlinear response are provided in Supplementary Note 4.

### Stochastic activation

Inside the bistable region, natural thermal noise can activate spontaneous transitions between low- and high-amplitude states. However, in high vacuum these events are extremely rare, which makes our system a promising candidate for nonlinear sensing. To observe stochastic activation within a reasonable time we therefore add to the EOM a Gaussian noise *ζ*(*t*) of amplitude *N*_*V*_, that in turns produces a position dependent parametric stochastic force of the form 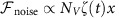. Noise calibration can be performed by looking at the particle's oscillation amplitude *A* in the absence of parametric driving^23^. For low noise levels, the particle's dynamics remains unaffected, being still dominated by the thermomechanical noise of the environment. However, for sufficiently high noise, the particle's amplitude starts to increase. Assuming an effective temperature 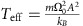, the noise amplitudes *N*_*V*_ are calibrated into meaningful temperature units *T*_N_ (Supplementary Note 5). Interestingly, the measured trend differs from what reported in other systems^8^, where a quadratic dependence between *T*_*N*_ and *N*_*V*_ was found. This is to be ascribed to the parametric (that is, multiplicative) nature of the noise injected in the present case, and prompts the interest of future studies.

**Figure 2:**
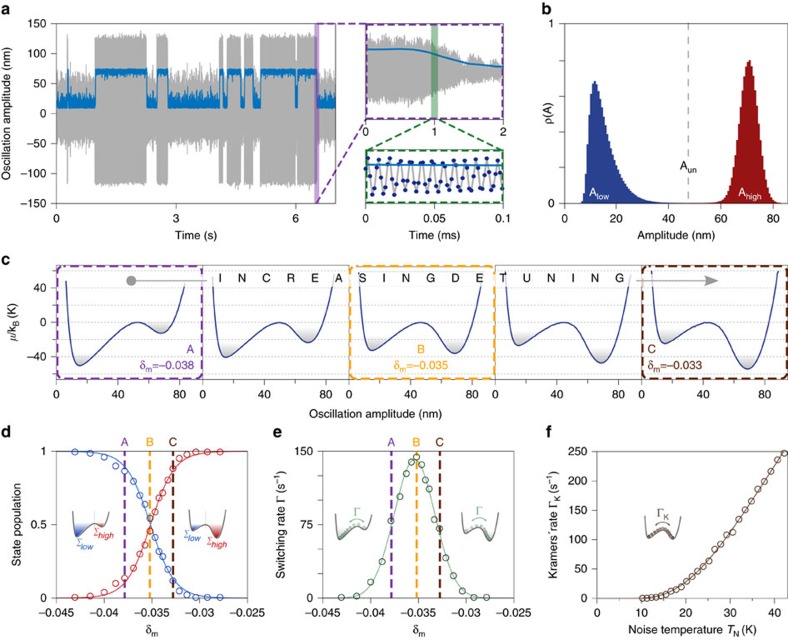
Stochastic switching and effective potential shaping. (**a**), Position time trace (grey) and corresponding amplitude (blue) reveal a typical bistable behaviour of the system, with randomly distributed stochastic switches between the two stable oscillation states. The distinctive trait of our system is to be able to follow the dynamics of the overdamped variable down to very short time scales (insets) and therefore control the dynamics with high accuracy. (**b**) A histogram of the amplitude distribution featuring two fully separated amplitude states from which the effective potential 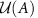 can be retrieved by inverting the Boltzmann-Gibbs distribution. (**c**) With *ad-hoc* tuning of the driving parameters 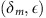 the potential can be finely shaped. For the dynamics here shown one expects a state population inversion and a maximization of the transition rate as shown in **d** and **e** respectively, where points A, B and C correspond to the marked potentials in **c**. (**f**) Transition rate as a function of noise injected for the symmetric potential case. Solid line is a fit to Kramers' law, which yields Γ_0_=(1,814±96) s^−1^ and 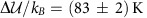.

The stochastic switching dynamics of the levitated nanoparticle is investigated via the injection of noise. Figure 2a illustrates position and amplitude time traces corresponding to few switching events and emphasizes the remarkable ability of our tracking scheme to resolve considerably different time scales. The amplitude probability distribution *ρ*(*A*), obtained by analysing the particle trajectory over ∼1 min, is shown in Fig. 2b. Even though the particle is in a non-thermal state due to the applied parametric control^13^, we introduce a simplified model that describes the dynamics of its amplitude as the motion of a thermal fictitious particle in a double well potential. This justifies the use of the Boltzmann–Gibbs distribution 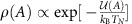 to extract the amplitude effective potential 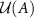 that models the bistable dynamics. Note that this approximation does not induce any inconsistency in the subsequent analysis, which relies in the experimental determination of the symmetric condition for 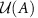. The high-frequency stability of the system allows us to finely modify the probability distribution *ρ*(*A*), and hence to engineer almost at will the shape of 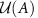 following any path inside the bistable regime. An interesting case consists of the so called iso-amplitude lines: paths along which the amplitude 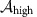, and consequently the amplitude gap 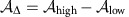, is kept constant. These particular subsets of the phase space are nonlinear features generally hidden by the frequency noise that blurs the measured instability tongue^10^,^21^. Thanks to the reduced frequency fluctuations, they are clearly visible in our system. Few examples of iso-amplitude lines are shown in the lower panel of Fig. 1d (solid lines), and satisfy the following relation (See Supplementary Note 4 for derivation):


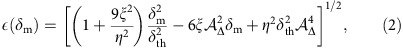


being 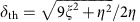. By following an iso-amplitude line with a fixed 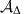, we obtain a very smooth evolution of the effective potential (Fig. 2c). Starting from an asymmetrically tilted configuration, it progressively undergoes inversion of its shape, passing through a quasi-symmetric condition where the potential barrier determines equal depth of the two wells. It is important to stress that, upon this *ad-hoc* dynamical sweep of 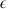 and *δ*_*m*_, the two minima of the potential corresponding to the amplitudes 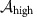 and 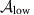, maintain their position fixed. Clearly, this would not be the case when following any other path, for example moving along 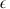
=const lines (see cross-cuts in Fig. 1d). The populations of the two states Σ_high_ and Σ_low_, defined as the normalized integrals of the amplitude distribution respectively above and below a threshold 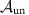, are shown in Fig. 2d, and display an inversion consistent with the potential dynamics observed in Fig. 2c. The switching rate Γ also depends significantly on the detuning (Fig. 2e), with a maximum corresponding to the symmetric potential configuration. Under this symmetry condition the switching rate, as a function of noise temperature *T*_*N*_, is expected to follow Kramers' law 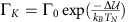. However, the injection of noise and its interplay with nonlinearities leads to a drift of the oscillation frequency, equivalent to a change in the detuning *δ*_m_, thereby distorting the symmetric potential (see Supplementary Note 6). If this change were not taken into account, a decay of the switching rate would be observed^8^. Conversely, if an adjusted detuning 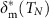 is applied (Supplementary Fig. 8), the symmetric potential is preserved and the prediction of Kramers' law is perfectly met, as we show in Fig. 2f.

### Stochastic resonance

The achieved control over the bistable dynamics makes our system a very flexible platform for studying two different signal amplification schemes. The first relies on a common phenomenon in bistable systems called stochastic resonance (SR)^7^. A weak periodic perturbation induces a modulation of the potential barrier separating the two states, and leads to an overall synchronized (that is, quasi-coherent) switching dynamics when the interwell transition rate matches twice the frequency of the perturbation. Given that this rate depends monotonically on the amount of noise in the system, SR leads to a noise-induced rise (and then fall) of the signal-to-noise ratio (SNR), and can therefore be exploited to amplify a narrow-band signal in a nonlinear system under appropriate conditions.

To this aim, we prepare the bistable system driving the particle with suitably chosen parameters 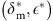. Once more, the noise-dependent detuning 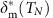 avoids the escape from the bistable region as a consequence of hysteresis quenching^8^,^24^, which in turn would prevent the observation of the full SR curve when noise is increased. The periodic (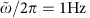) perturbation is then introduced modulating the depth of the parametric driving signal, namely 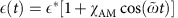, where the modulation strength *χ*_AM_ corresponds to optical forces of ten to a hundred atto-Newtons for the parameters used in our experiment. Amplitudes are measured with long acquisition times (∼10^3^ s) and repeated for increasing noise temperatures.

**Figure 3:**
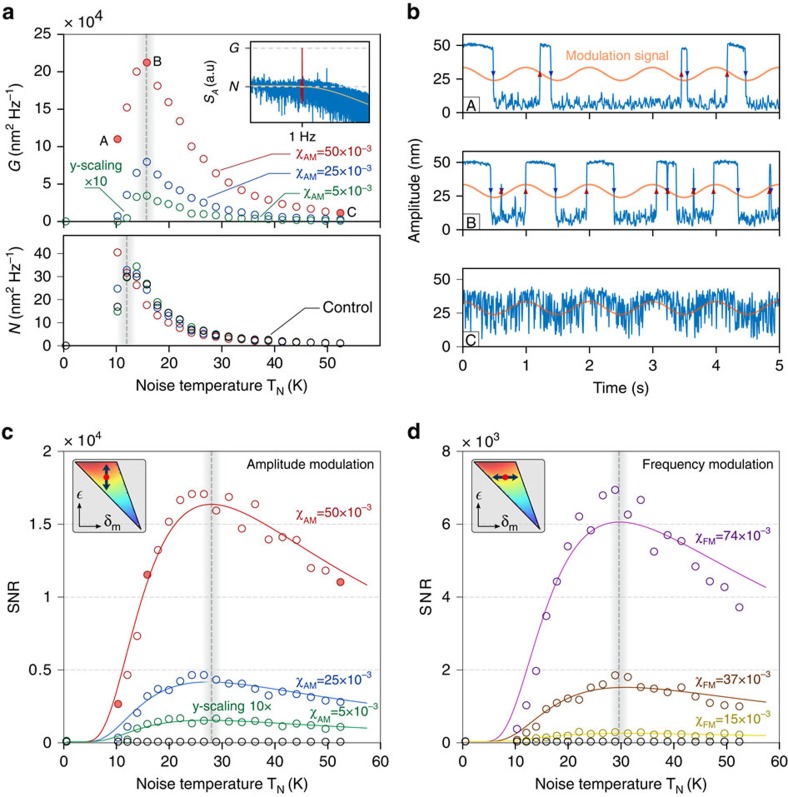
Stochastic resonance experiment. (**a**) Spectral amplification *G* and noise floor *N* as a function of the noise temperature for three different modulations. (**b**) Three examples of amplitude time traces for different noise temperatures (corresponding to points A,B and C in **a**), together with the modulation signal (orange line, not to scale in the *y* axis). For low noise (A) there is little correlation between switching dynamics and modulating signal. However, for higher noise (B) the system reaches synchronization and a maximum in *G* is found. If further increased (C), the noise leads to a degradation of the coherence in the switching dynamics. (**c**) The signal to noise ratio (SNR) of the detected modulating signal clearly presents a maximum (resonance) at *T*_*N*_∼28 K. Note that the peak position depends on both *G* and *N* and is therefore not expected to coincide with the peak in **a**. (**d**) The SNR curve for the SR experiment performed with frequency modulation of the driving signal. The same observations reported for **c** apply here. In both cases, black circles represent control data with *χ*_*AM*/*FM*_=0 to show that no amplification is encountered without modulation.

At *T*_*N*_∼10 K (point marked A) switching events are only partially correlated with the modulation signal (Fig. 3b). Instead, when noise is increased to *T*_*N*_∼16 K (point B) the system clearly exhibits an overall synchronization. A spectral analysis of the corresponding amplitude trace shows that *S*_*A*_ features an extremely sharp peak precisely at 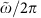 (see inset in Fig. 3a), for which spectral amplification *G* and noise floor *N* can be defined. When noise is further increased, the switching dynamics loses correlation with the modulation signal. Interestingly, a similar and counterintuitive behaviour is observed in the noise floor *N*, which presents a similar non-monotonic trend caused by a redistribution of noise intensity towards higher frequencies. Linear perturbation theory of stochastic resonance^7^ (Supplementary Note 7) predicts a *SNR*=*G*/*N* of the form:


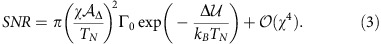


where *χ* is the modulation of the potential, proportional to *χ*_*AM*_.

Figure 3c shows the experimental SNR curves for different values of *χ*_AM_, together with a fitting of equation (3) that displays very good agreement. Similarly, the ratios of the fitted modulation depths (2: 5.4: 11) are also in good agreement with the expected ratios (2: 5: 10).

The second SR experiment was carried out for frequency modulation of the driving signal, 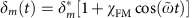, which according to our study (Fig. 2c), induces a potential modulation consistent with SR requirements. The corresponding results are shown in Fig. 3d. We emphasize the fact that previous experiments have only explored amplitude modulation, and that this is the first experimental demonstration of SR with frequency modulated signals. Again the fitted modulation strengths give ratios (1.9: 2.6: 4.9), in good agreement with the expected ones (2: 2.5: 5). Interestingly, the resonance appears at a noise temperature equivalent to the one observed in the amplitude modulation case. This is consistent with the fixed value of 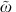 along the two experiments that requires an equal Γ_*K*_ in order to fulfil the time-scale matching of the resonant condition.

### Direct amplification

**Figure 4:**
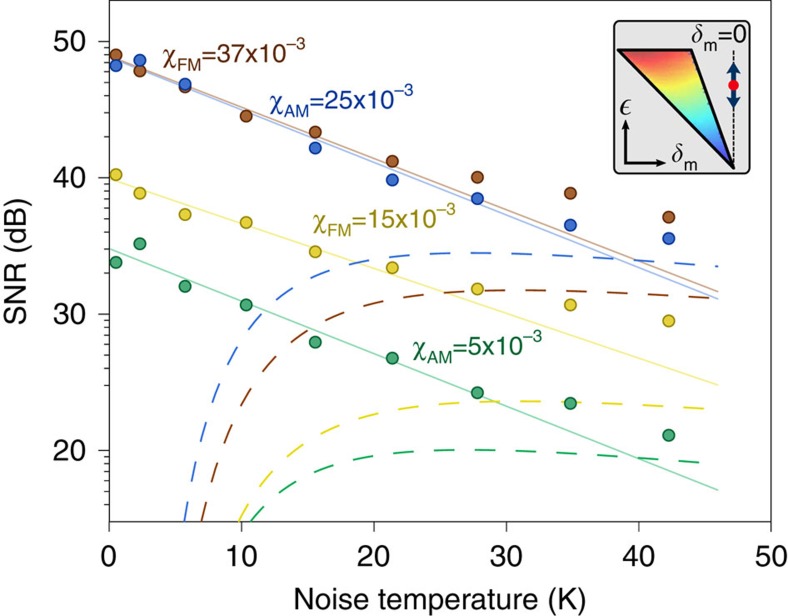
Direct amplification experiment. SNR of a 1 H*z* weak modulation signal detected by the system prepared at resonance (*δ*_m_=0) outside of the bistable regime, but still inside the instability tongue (inset). Circles are experimental data points for different AM/FM runs. Solid lines are fitted exponential decays, while dashed lines correspond to the fitted functions (3) of Fig. 3c,d and allow a straight comparison of the results obtained with the SR experiment (colour coding preserved). In particular, independently of the noise level, direct amplification always features a higher SNR than the corresponding SR case, with the two curves approaching for high noise, in agreement with the central dogma of signal detection theory.

Our platform enables us to directly compare the SR experiment with another amplification scheme that is implemented by changing the driving parameters to zero detuning. For *δ*_*m*_=0, the particle is driven outside of the bistable regime, but still inside the instability tongue (see inset in Fig. 4). In this configuration, the effective potential is monostable, and the AM and FM modulations applied (having same *χ*_*AM*/*FM*_ as in the SR case) result in a modulation of the oscillation amplitude for which a spectral analysis still leads to the observation of a peak at 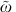. The corresponding SNR as a function of noise temperature *T*_*N*_ are shown in Fig. 4, together with fitted exponential decays that properly follow the experimental data for *T*_*N*_<30 K, and the fits of Fig. 3c,d in order to ease a direct comparison of the two amplification schemes. Independently of the noise level, direct amplification always features a higher SNR than the corresponding SR, and the two methods give similar outcomes at high *T*_*N*_. This result is an experimental verification of the central dogma of signal detection theory^25^, namely that stochastic resonance can decrease the SNR degradation of a noisy signal but it does not provide a mechanism by which the undetectable becomes detectable^26^,^27^. The couterinuitive nature of SR, in fact, mainly relies on suboptimal parameter ranges^28^, here exemplified by the fact that the same modulation becomes remarkably detectable when the detuning is set to zero, without the need of adding noise. Nonetheless, the fact of SR being observed in a wide variety of settings poses the question of why this phenomenon is omnipresent and favoured by nature. We surmise that suboptimal balanced configurations are generally preferable when dealing with very complex systems, for which optimal conditions would be difficult to achieve. Thus, exploiting ambient noise and stochastic resonance appears to be a successful strategy to ensure a robust amplification method that, at least above certain thresholds, is less sensitive to noise changes than other detection schemes (Fig. 4).

## Discussion

In conclusion, we presented an extensive study of the stochastic bistable dynamics of a levitated nanoparticle in high vacuum. The high stability achieved—in particular close to the bifurcation point—enabled the application of our system as a test platform for different amplification schemes, leading (to our knowledge) to the first qualitative and quantitative agreement between the stochastic resonance of a nanomechanical resonator and the corresponding analytical models. The present state of the experiment allowed up to ∼50 dB amplification of a parametric force at the attonewton scale that was mimicked by modulating the trap stiffness. A similar perturbation could arise from a time varying nonlinear potential. However, to evaluate the performance of our system to detect a conventional linear force, a direct actuation is required. This could be implemented by electrically coupling a charged particle^29^ to an external field^12^ or via scattering force from a weakly focused beam and is the subject of future work. The unprecedented performances demonstrated could enable the realization of novel ultra-sensitive threshold sensors, capable of detecting tiny perturbations via a state change in the system, or other detection schemes based on nonlinear nanomechanical resonators^3^,^4^. Likewise, a high-Q parametrically driven Duffing resonator could boost the state-of-the-art in nanomechanical memory elements^30^,^31^ introducing additional bits by simultaneous manipulation of the orthogonal oscillation modes^32^. In the parametrically driven regime our system could also enable fundamental search for classical to quantum transitions^33^, and prompt the realization of quantum-enhanced sensing techniques. Finally, we foresee in optically levitated nanoparticles a suitable platform for mimicking very complex stochastic nonlinear dynamics, able to shine a light on natural phenomena such as bio-molecule folding^16^,^17^, hearing^34^,^35^ and neural signalling^36^.

## Methods

### Sampling

The amplitude 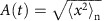 is tracked by sampling position *x* at *f*_*s*_=625 kHz and integrating over *n* successive positions measurements. The value of *n* depends on the temporal resolution needed in the particular measurement to be carried out. We set *n*=8,192 for the data shown in Fig. 1, *n*=128 for the amplitude time traces (blue data) of Fig. 2a and to *n*=4,096 for the SR experiment of Fig. 3. In this latter data sets, the acquisition time of a single amplitude trace at constant noise temperature (Fig. 3b) was 10^3^ s. However, in order to display a meaningful dynamics, we show only 5 s time traces.

### Data availability

The data that support the findings of this study are available from the corresponding author on reasonable request.

## Additional information

**How to cite this article:** Ricci, F. *et al*. Optically levitated nanoparticle as a model system for stochastic bistable dynamics. *Nat. Commun.* **8**, 15141 doi: 10.1038/ncomms15141 (2017).

**Publisher's note:** Springer Nature remains neutral with regard to jurisdictional claims in published maps and institutional affiliations.

## Supplementary Material

Supplementary Information Supplementary Figures, Supplementary Notes and Supplementary References.

Peer Review File

## References

[b1] Gieseler J., Novotny L. & Quidant R. Thermal nonlinearities in a nanomechanical oscillator. Nat. Phys. 9, 806–810 (2013).

[b2] Villanueva L. G. . Surpassing fundamental limits of oscillators using nonlinear resonators. Phys. Rev. Lett. 110, 177208 (2013). 23679770 10.1103/PhysRevLett.110.177208 PMC3839326

[b3] Aldana S., Bruder C. & Nunnenkamp A. Detection of weak forces based on noise-activated switching in bistable optomechanical systems. Phys. Rev. A 90, 063810 (2014).

[b4] Papariello L., Zilberberg O., Eichler A. & Chitra R. Ultrasensitive hysteretic force sensing with parametric nonlinear oscillators. Phys. Rev. E 94, 022201 (2016). 27627292 10.1103/PhysRevE.94.022201

[b5] Karabalin R. B. . Signal amplification by sensitive control of bifurcation topology. Phys. Rev. Lett. 106, 094102 (2011). 21405626 10.1103/PhysRevLett.106.094102

[b6] Siddiqi I. . RF-driven Josephson bifurcation amplifier for quantum measurement. Phys. Rev. Lett. 93, 207002 (2004). 15600958 10.1103/PhysRevLett.93.207002

[b7] Gammaitoni L., Hänggi P. & Jung P. Stochastic resonance. Rev. Mod. Phys. 70, 223–287 (1998).

[b8] Venstra W. J., Westra H. J. R. & van der Zant H. S. J. Stochastic switching of cantilever motion. Nat. Commun. 4, 2624 (2013). 24177274 10.1038/ncomms3624

[b9] Badzey R. L. & Mohanty P. Coherent signal amplification in bistable nanomechanical oscillators by stochastic resonance. Nature 437, 995–998 (2005). 16222295 10.1038/nature04124

[b10] Almog R., Zaitsev S., Shtempluck O. & Buks E. Signal amplification in a nanomechanical Duffing resonator via stochastic resonance. Appl. Phys. Lett. 90, 013508 (2007). 10.1103/PhysRevLett.98.078103 17359065

[b11] Gieseler J., Deutsch B., Quidant R. & Novotny L. Subkelvin parametric feedback cooling of a laser-trapped nanoparticle. Phys. Rev. Lett. 109, 103603 (2012). 23005289 10.1103/PhysRevLett.109.103603

[b12] Ranjit G., Cunningham M., Casey K. & Geraci A. A. Zeptonewton force sensing with nanospheres in an optical lattice. Phys. Rev. A 93, 053801 (2016).

[b13] Gieseler J., Quidant R., Dellago C. & Novotny L. Dynamic relaxation of a levitated nanoparticle from a non-equilibrium steady state. Nat. Nanotechnol. 9, 358–364 (2014). 24681775 10.1038/nnano.2014.40

[b14] Millen J., Deesuwan T., Barker P. & Anders J. Nanoscale temperature measurements using non-equilibrium Brownian dynamics of a levitated nanosphere. Nat. Nanotechnol. 9, 425–429 (2014). 24793558 10.1038/nnano.2014.82

[b15] Jain V. . Direct measurement of photon recoil from a levitated nanoparticle. Phys. Rev. Lett. 116, 243601 (2016). 27367388 10.1103/PhysRevLett.116.243601

[b16] Hayashi K., de Lorenzo S., Manosas M., Huguet J. M. & Ritort F. Single-molecule stochastic resonance. Phys. Rev. X 2, 031012 (2012).

[b17] Angeli D., Ferrell J. E. & Sontag E. D. Detection of multistability, bifurcations, and hysteresis in a large class of biological positive-feedback systems. Proc. Natl Acad. Sci. USA 101, 1822–1827 (2004). 14766974 10.1073/pnas.0308265100 PMC357011

[b18] Simakov D. S. A. & Pérez-Mercader J. Noise induced oscillations and coherence resonance in a generic model of the nonisothermal chemical oscillator. Sci. Rep. 3, 2404 (2013). 23929212 10.1038/srep02404 PMC3738947

[b19] Hänggi P., Talkner P. & Borkovec M. Reaction-rate theory: Fifty years after Kramers. Rev. Mod. Phys. 62, 251–341 (1990).

[b20] Myers C. J., Celebrano M. & Krishnan M. Information storage and retrieval in a single levitating colloidal particle. Nat. Nanotechnol. 10, 886–891 (2015). 26280408 10.1038/nnano.2015.173

[b21] Gieseler J., Spasenović M., Novotny L. & Quidant R. Nonlinear mode coupling and synchronization of a vacuum-trapped nanoparticle. Phys. Rev. Lett. 112, 103603 (2014). 24679293 10.1103/PhysRevLett.112.103603

[b22] Lifshitz R. & Cross M. C. Nonlinear Dynamics of Nanomechanical and Micromechanical Resonators 1–52Wiley-VCH (2009).

[b23] Mestres P., Martinez I. A., Ortiz-Ambriz A., Rica R. A. & Roldan E. Realization of nonequilibrium thermodynamic processes using external colored noise. Phys. Rev. E 90, 032116 (2014). 10.1103/PhysRevE.90.032116 25314404

[b24] Aldridge J. S. & Cleland A. N. Noise-enabled precision measurements of a duffing nanomechanical resonator. Phys. Rev. Lett. 94, 156403 (2005). 15904165 10.1103/PhysRevLett.94.156403

[b25] Tougaard J. Signal detection theory, detectability and stochastic resonance effects. Biol. Cybern. 87, 79–90 (2002). 12181584 10.1007/s00422-002-0327-0

[b26] Petracchi D. What is the role of stochastic resonance? Chaos Solitons Fractals 11, 1827–1834 (2000).

[b27] Dykman M. I. & McClintock P. V. E. What can stochastic resonance do? Nature 391, 344 (1998).9450746

[b28] McDonnell M. D. & Abbott D. What is stochastic resonance? Definitions, misconceptions, debates, and its relevance to biology. PLoS Comput. Biol. 5, e1000348 (2009). 19562010 10.1371/journal.pcbi.1000348 PMC2660436

[b29] Alda I., Berthelot J., Rica R. A. & Quidant R. Trapping and manipulation of individual nanoparticles in a planar paul trap. Appl. Phys. Lett. 109, 163105 (2016).

[b30] Mahboob I., Okamoto H. & Yamaguchi H. Two-mode thermal-noise squeezing in an electromechanical resonator. Phys. Rev. Lett. 113, 167203 (2014). 25361277 10.1103/PhysRevLett.113.167203

[b31] Bagheri M., Poot M., Li M., Pernice W. P. H. & Tang H. X. Dynamic manipulation of nanomechanical resonators in the high-amplitude regime and non-volatile mechanical memory operation. Nat. Nanotechnol. 6, 726–732 (2011). 22020123 10.1038/nnano.2011.180

[b32] Frimmer M., Gieseler J. & Novotny L. Cooling mechanical oscillators by coherent control. Phys. Rev. Lett. 117, 163601 (2016). 27792359 10.1103/PhysRevLett.117.163601

[b33] Katz I., Retzker A., Straub R. & Lifshitz R. Signatures for a classical to quantum transition of a driven nonlinear nanomechanical resonator. Phys. Rev. Lett. 99, 040404 (2007). 17678342 10.1103/PhysRevLett.99.040404

[b34] Eguíluz V. M., Ospeck M., Choe Y., Hudspeth A. J. & Magnasco M. O. Essential nonlinearities in hearing. Phys. Rev. Lett. 84, 5232–5235 (2000). 10990910 10.1103/PhysRevLett.84.5232

[b35] Martignoli S., Gomez F. & Stoop R. Pitch sensation involves stochastic resonance. Sci. Rep. 3, 2676 (2013). 24045830 10.1038/srep02676 PMC3776232

[b36] McDonnell M. D. & Ward L. M. The benefits of noise in neural systems: bridging theory and experiment. Nat. Rev. Neurosci. 12, 415–426 (2011). 21685932 10.1038/nrn3061

