# Supplementary material for: Optically levitated nanoparticle as a model system for stochastic bistable dynamics
Source: Nat Commun. 2017 May 9;8:15141. doi: 10.1038/ncomms15141 (PMC5436086; doi:10.1038/ncomms15141)
Supplement: Supplementary Information — Supplementary Figures, Supplementary Notes and Supplementary References. [file ncomms15141-s1.pdf]

SUPPLEMENTARY NOTE 1: Detailed experimental set-up

The full experimental set-up is schematically shown in Fig. 1. The light source is a an ultra-stable low noise Nd:YAG laser, and is used to both trap the silica nanoparticle ($d \sim 177$nm in diameter) and track its motion. After passing a Faraday isolator (FI), the beam is split into a high power branch (trapping beam) and a low power branch (detection beam) by means of a polarizing beam splitter (PBS). To decouple the two branches and to avoid interferences in the laser focus we use cross polarized beams and we shift the frequency ($\Delta f \approx 110$MHz) of the detection beam with an acousto-optic modulator (AOM). The trapping beam passes through the electro-optic modulator (EOM) that allows the parametric actuation of the particle. After convenient beam shaping with telescope systems (TS) the two paths are recombined with a second PBS before entering in the vacuum chamber where a high NA microscope objective (OBJ) focuses the beams - creating the optical trap - and an aspheric lens (AL) collects and collimates the light, further sent out of the chamber carrying the information about the particle's motion. After the vacuum chamber the trapping beam is dumped, while the detection beam is analysed with a split detection scheme. This scheme relies on the fact that the interference pattern between the transmitted light and the light scattered from the particle is position dependent. Therefore, by half-splitting the beam with a sharp edge D-shaped mirror (DM) and sending the two halves to a balanced photodetector, it is possible to measure the oscillating component of the input signal, which is proportional to the particle's position. Figure 4(a) shows the ability of such a scheme to track particle position with a noise floor on the order of 1 pm/$\sqrt{\text{Hz}}$ [1].

Below ~ 6 mbar pressures, the particle enters in the ballistic regime, and a spectral analysis of its motion shows the presence of three peaks at the eigenfrequencies of the oscillation modes. Further decreasing the pressure we reach an highly underdamped regime ($P \simeq 10^{-6}$ mbar), where the particle oscillates with very small amplitude and corresponding sub-kelvin effective temperatures. At these temperatures the three mechanical modes of the oscillation are fully decoupled and the restoring gradient force depends linearly on particle's displacement by means of the linear trap stiffness $\kappa_i = \alpha E_0^2/w_i^2$, where α_0 is the polarizability of SiO_2, E_0 is the electric field intensity at the focus and w_i is the beam waist radius ($i = x, y$) or Rayleigh range ($i = z$). Due to the asymmetry of the trapping potential, the three mechanical modes along the three main axes have different oscillation frequencies: $(\Omega_x, \Omega_y, \Omega_z)/2\pi \simeq (125, 140, 40)$ kHz, as we show in Fig. 2. Under this non-degentrate conditions, the feedback scheme can therefore act without cross-coupling between the three signals.

In the absence of feedback cooling, or for coherent driving of one of the modes, the amplitude of the oscillation becomes large and the particle explores the anharmonic part o the optical potential, which features Duffing nonlinearities. In this regime, the restoring force is well approximated by $F_i^{\text{grad}} = -\kappa_i \left(1 + \sum_{j=x,y,z} \xi_j x_j^2\right) x_i$, where x_i is the displacement of the particle from the trap centre and the nonlinear coefficients are given by $\xi_j = -2/w_j^2$ [2]. The coefficients ξ_j can be calculated with high accuracy measuring the particle motion in the nonlinear oscillation regime, as explained in the main text and shown in Fig. 1d.

SUPPLEMENTARY NOTE 2: Feedback electronics optimization

The high stability of our system takes partially merit from a fine optimization of the feedback electronics, with particular regards to the *phase shifter and frequency doubler* module, responsible for the implementation of an efficient cooling. In fact the relative phase between particle oscillation and feedback signal is a crucial parameter: a phase shift of $\pi/2$, for example, turns cooling (nonlinear

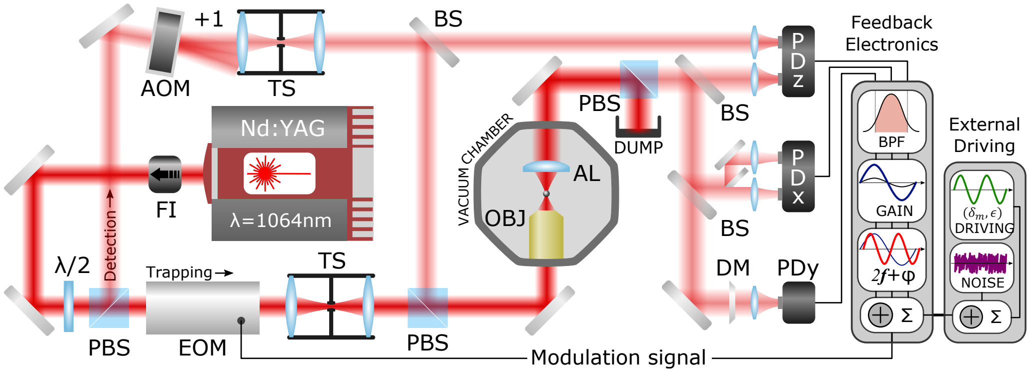

Supplementary Figure 1: **Optical set-up and feedback electronics.** Full schematics of the experimental set-up. Detailed description of set-up components is provided in the Supplementary Section 1 text

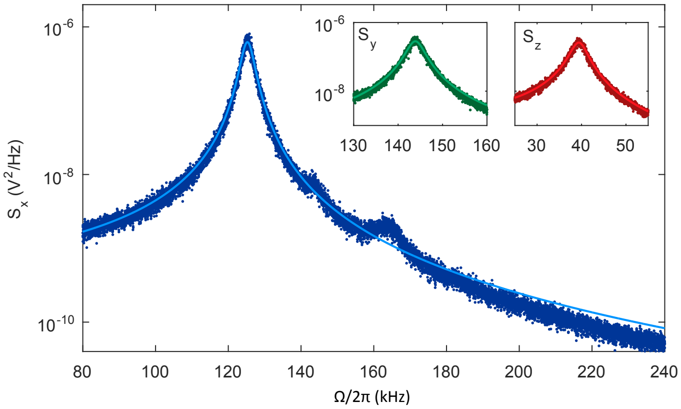

Supplementary Figure 2: **Power spectral densities of the decoupled modes** Experimental power spectral densities S_i $(i = x, y, z)$ of the particle's 3D motion at $P \sim 6$ mbar (dots), and corresponding Lorentzian fitted function (solid lines). The peaks appear at non-degenerate frequencies, which allows the feedback to act separately on each oscillation mode. Also, decoupling is ensured in our system, as almost no trace of orthogonal oscillation is present in each considered mode.

damping term $\eta > 0$) into amplification ($\eta < 0$). Therefore, a fine tuning of the feedback phase is of uttermost importance. As explained in the main text, one way to retrieve the value of the nonlinear damping coefficient η is to measure and fit the nonlinear amplitude response of the particle during parametric driving frequency *down-sweeps*, which reads:

$$A^2(\delta_{\mathrm{m}}, \epsilon; \eta, \xi) \approx \frac{1}{\eta \delta_{\mathrm{th}}^2} \left[3\frac{\xi}{\eta}\delta_{\mathrm{m}} + \sqrt{\epsilon^2 \delta_{\mathrm{th}}^2 - \delta_{\mathrm{m}}^2} \right] \tag{1}$$

Fig. 3 sketches the procedure that allows to optimize the feedback efficiency for the three channels (oscillation modes) separately. For each value of phase ϕ we measure the particle response inside the Arnold tongue (3(a)). Then, by fitting the oscillation amplitude at different ϵ values (3(b)), and discarding the fits with an *R-square* lower than a certain threshold (=0.995 for the case in Fig. 3(c)) we retrieve η and the relative standard deviation σ_η from a weighted averaging over the fits outcomes. The procedure is repeated for each mode x, y, z. We clearly from Fig. 3(d) that the non-linear damping coefficients ($\eta_{\mathrm{x}}, \eta_{\mathrm{y}}, \eta_{\mathrm{z}}$) display a maximum in their trend, which consequently provides the optimal phase delays (ϕ_x, ϕ_y, ϕ_z) to be set.

SUPPLEMENTARY NOTE 3: Frequency stability

Frequency stability of the resonator is monitored by tracing the resonance peak position in time. Examples of relative frequency time traces are shown in Fig. 4(b). At high pressure, and with no feedback (purple trace), relative fluctuations are of the order of $\sigma_\Omega/\Omega_0 \approx 10^{-2}$. However when high vacuum is reached and the feedback cooling is turned on (orange trace), we find $\sigma_\Omega/\Omega_0 \approx 10^{-4}$, and a net fluctuation reduction of two orders of magnitude is observed. The remarkably small values obtained correspond, in absolute terms, to fluctuations of ~ 30 Hz, smaller than the effective linewidth of the resonator (~ 100 Hz, considering feedback broadening effects). The considerable improvement in frequency stability, as compared to previous experiment [2, 3], has been achieved by reducing the two main sources of disturbances, namely the optical noise introduced by the feedback cooling, and air turbulences affecting pointing stability of both the trapping beam and the detection scheme. A comprehensive description of feedback optimization is given in Supplementary SUPPLEMENTARY NOTE 2:, while air disturbances problems have been circumvented by hardware screening the optical set-up.

SUPPLEMENTARY NOTE 4: Nonlinear response

Parametrically driving the particle into the nonlinear regime leads to an expected amplitude response given by [3, 4]

$$A^2(\delta_{\mathrm{m}}, \epsilon; \eta, \xi) \approx \frac{1}{\eta \delta_{\mathrm{th}}^2} \left[3\frac{\xi}{\eta}\delta_{\mathrm{m}} + \sqrt{\epsilon^2 \delta_{\mathrm{th}}^2 - \delta_{\mathrm{m}}^2} \right] \tag{2}$$

Performing frequency *down-sweeps* and *up-sweeps* also provides interesting spectral features of the two oscillation modes. These are shown in Fig. 5a. The low amplitude mode $\mathcal{A}_{\mathrm{low}}$ consists of a frequency pulled main mode at Ω_{P}, together with the drive sideband at Ω_{S}. The position of these peaks depends of the driving parameters ($\delta_{\mathrm{m}}, \epsilon$) according to the relation $\Omega_{\mathrm{P,S}} = \Omega_0 \left[1 + \frac{\delta_{\mathrm{m}} \pm \sqrt{\delta_{\mathrm{m}}^2 - (\epsilon/2)^2}}{2} \right]$, with the $\pm$ sign corresponding to P and S subscripts respectively. Instead, the high amplitude mode $\mathcal{A}_{\mathrm{high}}$ is a locked steady state oscillation, with fixed frequency $\Omega_0 (1 + \delta_{\mathrm{m}}/2) = \Omega_m/2$. These frequency relations along up- and down- sweeps are depicted in Fig. 5b, where we show experimental data (dots), together with analytical prediction (solid lines). These comparisons, namely both

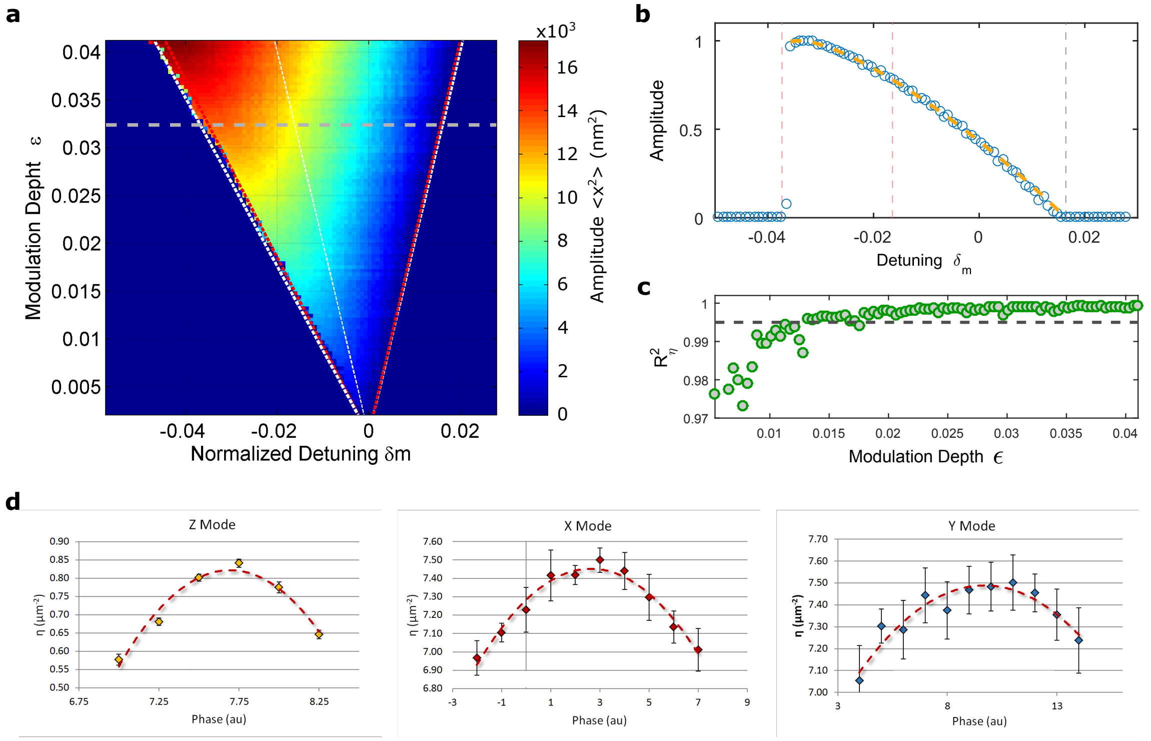

Supplementary Figure 3: **Feedback optimization procedure**. **(a),** 2D map of the amplitude response to a frequency down-sweep of the parametric driving. **(b),** Each horizontal cross cut is fitted with the analytical prediction (2), retrieving an estimation of the fit free parameters η and ξ. **(c),** The fitting error results very small, and a 0.995 threshold level on the determination coefficient R^2 allows to consider only fitted parameters in high agreement with the model. **(d),** Actual feedback optimization plots for the three eigenmodes. Setting the feedback phase (here displayed in arbitrary units,) to a proper value, leads to maximization of the nonlinear damping (i.e. cooling efficiency) of the system and a consequent optimization of the system performances.

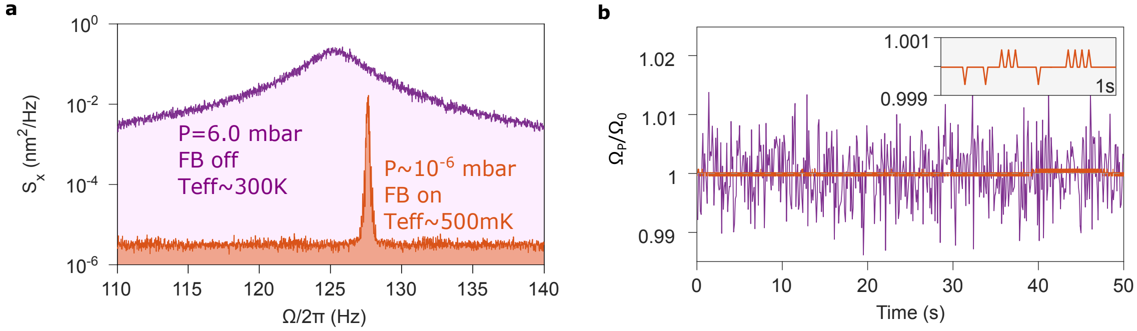

Supplementary Figure 4: **Feedback cooling spectral analysis and oscillation tracking**. **(a),** Position power spectral density of the x mode at different pressures. At $P \sim 6$ mbar thermal equilibrium conditions allow to calibrate the detection that we use in high vacuum ($P \sim 10^{-6}$ mbar) to perform the experiments. The feedback cooling provides a temperature compression of a factor ~ 600. **(b),** The trap stability is monitored looking at the instantaneous particle frequency in time. The FB cooling reduces the fluctuations by two orders of magnitude, and maintains the particle frequency within ~ 30 Hz deviation for trapping times of several months.

amplitude fits and iso-amplitude lines of Fig. 1d (main text), and the frequency curves in Fig. 5c demonstrate the high level of accuracy of our measurements and the perfect agreement with the model given by Eq. (1) of the main text.

The analytical prediction of the *iso-amplitude* lines shown in Fig. 1d (main text) directly follows from eq. 2 after representing the amplitude response as a 2-dimensional surface in the $(\delta_{\rm m}, \epsilon, A)$ space (cross-cuts in Fig. 1d actually give a hint of such a surface). By setting $A^2(\delta_{\rm m}, \epsilon) = \mathcal{A}_\Delta^2 = const$, one selects an horizontal cut of the surface, having the property of maintaining a constant amplitude , that projected in the $(\delta_{\rm m}, \epsilon)-$plane identifies the iso-amplitude line. Mathematical derivation is as follows:

$$
\begin{aligned}
A^2(\delta_{\rm m}, \epsilon) = \mathcal{A}_\Delta^2 = \text{const} \qquad & \rightarrow \quad \frac{1}{\eta\delta_{\rm th}^2}\left[3\frac{\xi}{\eta}\delta_{\rm m} + \sqrt{\epsilon^2\delta_{\rm th}^2 - \delta_{\rm m}^2}\right] = \mathcal{A}_\Delta^2 \\
& \rightarrow \quad \sqrt{\epsilon^2\delta_{\rm th} - \delta_{\rm m}^2} = \frac{1}{\eta}\left[\mathcal{A}_\Delta^2\eta^2\delta_{\rm th}^2 - 3\xi\delta_{\rm m}\right] \\
& \Rightarrow \quad \epsilon(\delta_{\rm m}) = \left[\left(1 + \frac{9\xi^2}{\eta^2}\right)\frac{\delta_{\rm m}^2}{\delta_{\rm th}^2} - 6\xi\mathcal{A}_\Delta^2\delta_{\rm m} + \eta^2\delta_{\rm th}^2\mathcal{A}_\Delta^4\right]^{1/2} \qquad (3)
\end{aligned}
$$

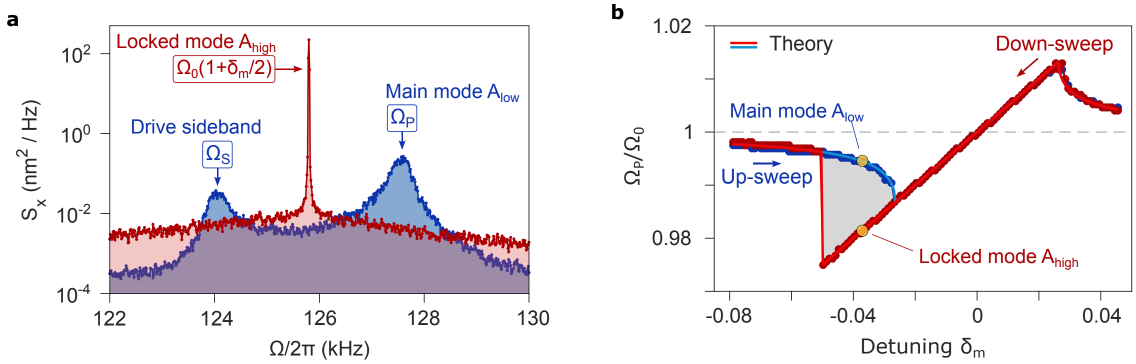

Supplementary Figure 5: **Spectral features of the amplitude states. a,** Power spectral density of the oscillation amplitude states $\mathcal{A}_{\rm high}$ and $\mathcal{A}_{\rm low}$. The former is locked at half the frequency of the parametric modulation $\Omega_m/2$ while the latter presents a frequency pulled main mode at Ω_P and a drive sideband at Ω_S. **b,** Particle frequency Ω_P along up- and down-sweep. The shaded region corresponds to the bistable regime where frequency pulling takes place. Solid lines are theoretical prediction of the frequency trends, showing high level accuracy of the experimental measurements.

SUPPLEMENTARY NOTE 5: Noise Calibration

The system behaviour after noise injection is shortly described in the main text and here supported by the graphic data in Fig. 6. The 1 MHz bandwidth Gaussian noise process $\zeta(t)$, used to drive the particle in the stochastic switching regime, satisfies $\langle\zeta(t)\rangle = 0$ and $\langle\zeta(t)\zeta(t')\rangle = \delta(t-t')$. When noise amplitude N_V is varied from 0 to 8 V, we obtain the discussed increase in oscillation amplitude that provides the noise temperature calibration $T_N \approx N_V^{1.44}$ (see Fig. 6a). The histogram in the inset shows the energy distribution of the particle derived from a sample of 10^5 measurements. The

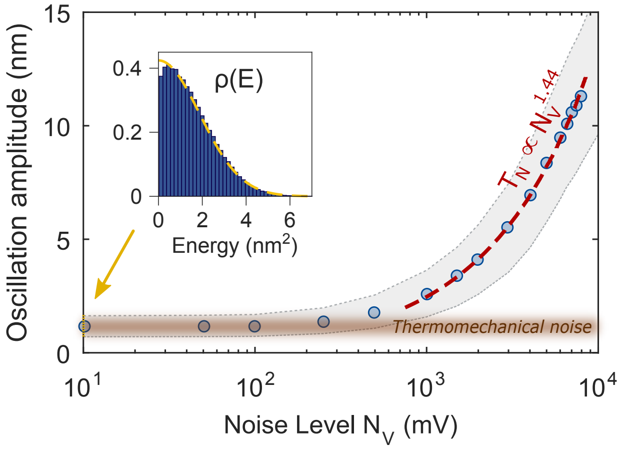

Supplementary Figure 6: **Noise temperature calibration without parametric driving. a**, When injecting optical noise in the system the particle's oscillation amplitude increases (blue dots), together with its standard deviation (shaded area). Converting amplitudes into effective temperatures it is possible to map noise levels into noise temperatures and extract a power law calibration curve. The inset shows the measured energy distribution $\rho(E)$ at the thermomechanical noise level.

dashed yellow line is a fitted analytical curve of the form:

$$\rho(E) \approx \exp\left[-\beta\left(E + \frac{\eta}{4m\Omega_0\gamma_0}E^2\right)\right] \tag{4}$$

where, given the high accuracy in the calculated value of η (see main text Fig. 1d), γ_0 remains the only fitting parameter.

Tracking the time evolution of the amplitude provides information about its spectral properties. By applying the *slow varying envelope approximation* to the equation of motion of the system in the absence of coherent driving (Eq. (1) main text, having set $\epsilon = 0$), one finds that the amplitude behaves as an overdamped variable, in spite of being the result of the average over a fast (underdamped) oscillating variable [3]. This behaviour is depicted in Fig. 6b where the amplitude power spectral density S_A is shown for different noise temperatures. We fit the measured power spectral densities (PSDs) with a typical overdamped Lorentzian $S_A = S_0\frac{f_c^2}{f^2+f_c^2}$ where $S_0 = S_A(f=0)$ is the height of the plateau and f_c is the corner frequency. The inset shows the power law dependency of these fitting parameters (in terms of relative change with respect to the no-injected-noise case) as a function of noise temperature. As expected, the low-frequency plateau raises with the noise intensity, in agrement with the observation in Fig. 6a [5, 6]. The corner frequency also increases with the injected noise, leading to a larger bandwidth of amplitude fluctuations, this likely being due to the fact that the optical noise becomes multiplicative after the EOM [7]

SUPPLEMENTARY NOTE 6: Symmetric effective potential

When measuring Kramers' transition rates a static symmetric configuration of the potential is needed. The same applies when performing the Stochastic Resonance (SR) experiment, where on top of a symmetric potential the harmonic perturbation contributes with a modulation of the shape and consequently of the switching probability. If such a starting symmetric condition is not

ensured, unwanted effects of probability quenching are encountered, and proper SR phenomena are not observed. Therefore, a preliminary fine characterization to know where this symmetry configuration is found inside the bistable regime is of utmost importance. To do so we sweep the detuning δ_{m} horizontally in a small region around this symmetry condition. During each (discretized) sweep we measure stochastic switching time traces and, identically to what explained in the main text, we retrieve the effective potential from from the amplitude histograms. Such a scan provides the data shown in Fig. 7. Fig. 7a,b show the amplitude of stable ($\mathcal{A}_{\mathrm{low}}$ and $\mathcal{A}_{\mathrm{high}}$) and unstable $\mathcal{A}_{\mathrm{un}}$ states, together with the amplitude gap $\mathcal{A}_{\Delta}$, as a function of the detuning δ_m. As the scan inspects only a narrow region of the bistable tongue the amplitude of the stable states, and consequently $\mathcal{A}_{\Delta}$ are kept constant, while the unstable state shift towards lower amplitudes due to the dynamical changes in the potential shape (see main text Fig. 2c). These changes are

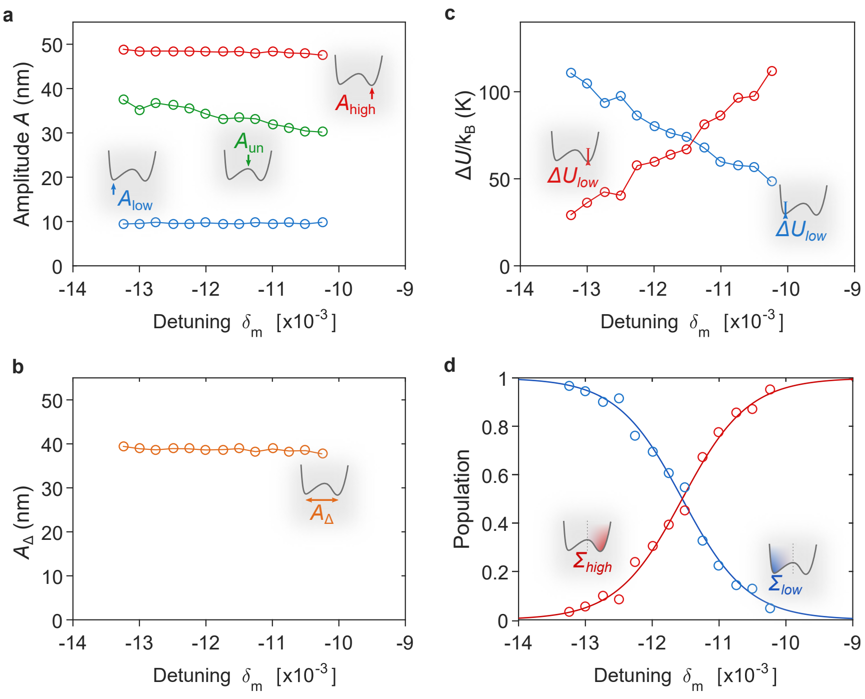

Supplementary Figure 7: **Symmetric potential configuration measurement**. **a,** Amplitude states $\mathcal{A}_{\mathrm{low}}$, $\mathcal{A}_{\mathrm{high}}$ and $\mathcal{A}_{\mathrm{un}}$ as a function of the scanned detuning. **b,** Over a narrow region of the bistable regime, the amplitude gap $\mathcal{A}_{\Delta}$ remains constant. **c,** Potential barrier height for $\mathcal{A}_{\mathrm{low}} \to \mathcal{A}_{\mathrm{high}}$ (blue) and $\mathcal{A}_{\mathrm{high}} \to \mathcal{A}_{\mathrm{low}}$ transitions (red). **d,** Similarly to what shown in Fig. 2d of the main text, state populations Σ_{low} and Σ_{high} invert at the symmetric configuration δ^*_{m}. As a result, fitting with sigmoid functions $\sigma_{\mathrm{low}}(\delta_{\mathrm{m}})$ and $\sigma_{\mathrm{high}}(\delta_{\mathrm{m}})$ (see eq. 5)provides information on the position δ^*_{m} of the symmetric configuration and on the width τ of the bistable regime.

also evident in Fig. 7c, where the hight of the potential barriers separating respectively the lower from the higher state and the vice versa, invert their values passing through the quasi-symmetric configuration. Correspondingly, the state populations in Fig. 7d also undergo a similar dynamics. The fitting function in the latter two plots are typical sigmoid function of the form

$$\sigma_{\mathrm{high}}(\delta_{\mathrm{m}}) = \frac{1}{1+\mathrm{e}^{-(\delta_{\mathrm{m}}-\delta^*_{\mathrm{m}})/\tau}} \quad ; \quad \sigma_{\mathrm{low}}(\delta_{\mathrm{m}}) = 1 - \frac{1}{1+\mathrm{e}^{-(\delta_{\mathrm{m}}-\delta^*_{\mathrm{m}})/\tau}} \tag{5}$$

where the parameter δ_m^* and τ respectively represent the horizontal position of the symmetric configuration inside the bistable tongue, and the effective width of the bistable regime. Repeating these measurements for higher levels of noise in the system yields to a clear dependency of these parameters from the noise temperature T_N. This trends are displayed in Fig. 8, where dots correspond to the value of δ_m^* and errorbars represent the width τ of the sigmoid. The outcome of

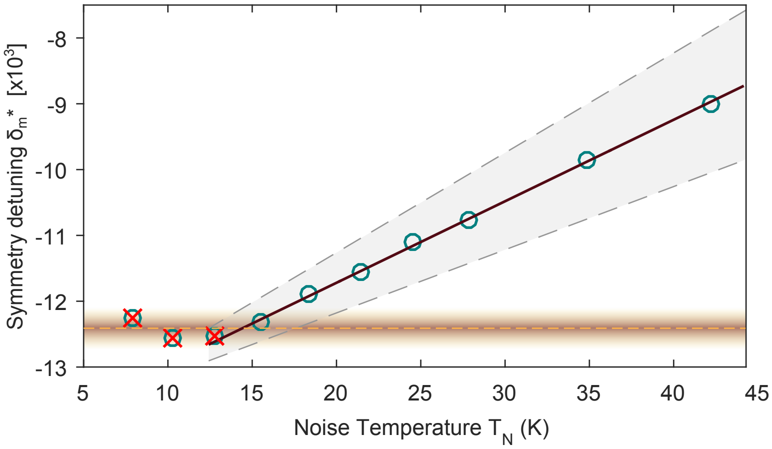

Supplementary Figure 8: **Symmetric potential configuration with noise temperature**. Values of $\delta_{\rm m}^*$ (circles) and width τ of the bistable regime (shaded region) as a function of noise temperature T_N. The linear fit (solid line) provides an intrinsic characterization of the system that allows to follow the symmetric potential configuration when higher levels of noise are injected. Red crosses correspond to data points excluded from the fit.

these measurements allows to conclude that when noise temperature is changed in the system, an adjustment to the driving parameters, and more precisely to detuning δ_m is necessary to lock on to the symmetric configuration. We believe the reason for this resides in two shift-contribution. On the one hand there is the noise-induced particle frequency drift (also observed in [8, 9]), which automatically requires the detuning to follow. On the other hand, instead, the multiplicative nature of the noise [7] could also play a role when entering in the fully noise-driven dynamics of the particle.

SUPPLEMENTARY NOTE 7: Linear perturbation theory of stochastic resonance

According to linear perturbation theory, the expected amplitude power spectral density (PSD) has the form:

$$S_A(\omega) = \left[1 - \frac{1}{2}\left(\frac{\chi\mathcal{A}_\Delta}{T_N}\right)^2 \frac{4\Gamma_K^2}{4\Gamma_K^2 + \tilde{\omega}^2}\right] \frac{4\Gamma_K \mathcal{A}_\Delta^2}{4\Gamma_K^2 + \omega^2} + \\ + \frac{\pi}{2}\left(\frac{\chi\mathcal{A}_\Delta}{T_N}\right)^2 \frac{4\Gamma_K^2 \mathcal{A}_\Delta^2}{4\Gamma_K^2 + \tilde{\omega}^2} \times \delta(\omega - \tilde{\omega}) \tag{6}$$

This equation represents the analytical prediction of the powr spectral density(PSD) shown in the inset of Fig. 3a (main text). Here the first term corresponds to the typical PSD of an overdamped variable, while the second one containing the δ-function is related to the harmonic perturbation. It is

interesting to note that if no modulation is present, i.e. $\chi = 0$, the spectral density reads $S_A(\omega) = \frac{4\Gamma_K \mathcal{A}_\Delta^2}{4\Gamma_K^2 + \omega^2}$ and still depends on the noise T_N via the Kramers rate Γ_K which is a monotonically increasing function of T_N. Consequently, the value of $S_A(\tilde{\omega})$, which in this case corresponds to the noise floor N, also features a maximum on its dependence with the noise injected in the system (check lower panel of Fig. 3a, main text). Due to the small values of χ, the noise floor shows the same behaviour for the modulated and the unmodulated (control) cases, according to eq. Eq. (6).

Supplementary References

1. Gieseler, J., Deutsch, B., Quidant, R. & Novotny, L. Subkelvin parametric feedback cooling of a laser-trapped nanoparticle. *Phys. Rev. Lett.* **109,** 1–5 (2012).
2. Gieseler, J., Novotny, L. & Quidant, R. Thermal nonlinearities in a nanomechanical oscillator. *Nat. Phys.* **9,** 806–810 (2013).
3. Gieseler, J., Spasenović, M., Novotny, L. & Quidant, R. Nonlinear mode coupling and synchronization of a vacuum-trapped nanoparticle. *Phys. Rev. Lett.* **112,** 1–5 (2014).
4. Lifshitz, R. & Cross, M. C. in *Reviews of Nonlinear Dynamics and Complexity* 1–52 (Wiley-VCH, 2009). ISBN: 9783527626359. doi:10.1002/9783527626359.ch1.
5. Martínez, I. A., Roldán, E., Parrondo, J. M. R. & Petrov, D. Effective heating to several thousand kelvins of an optically trapped sphere in a liquid. *Phys. Rev. E* **87,** 032159 (3 2013).
6. Mestres, P., Martinez, I. A., Ortiz-Ambriz, A., Rica, R. A. & Roldan, E. Realization of nonequilibrium thermodynamic processes using external colored noise. *Phys. Rev. E* **90,** 032116 (3 2014).
7. Volpe, G. & Wehr, J. Effective drifts in dynamical systems with multiplicative noise: a review of recent progress. *Rep. Prog. Phys.* **79,** 053901 (2016).
8. Aldridge, J. S. & Cleland, A. N. Noise-enabled precision measurements of a duffing nanomechanical resonator. *Phys. Rev. Lett.* **94,** 5–8 (2005).
9. Venstra, W. J., Westra, H. J. R. & van der Zant, H. S. J. Stochastic switching of cantilever motion. *Nat. Commun.* **4,** 2624 (2013).
