# Supplementary material for: Optically levitated nanoparticle as a model system for stochastic bistable dynamics
Source: Nat Commun. 2017 May 9;8:15141. doi: 10.1038/ncomms15141 (PMC5436086; doi:10.1038/ncomms15141)
Supplement: Peer Review File [file ncomms15141-s2.pdf]

Editorial Note: this manuscript has been previously reviewed at another journal that is not operating a transparent peer review scheme. This document only contains reviewer comments and rebuttal letters for versions considered at Nature Communications.
